# SiRNA Targeting mTOR Effectively Prevents the Proliferation and Migration of Human Lens Epithelial Cells

**DOI:** 10.1371/journal.pone.0167349

**Published:** 2016-12-02

**Authors:** Chunmei Zhang, Jingjing Liu, Na Jin, Guiming Zhang, Yahui Xi, Hongling Liu

**Affiliations:** 1 Department of Ophthalmology, the First Affiliated Hospital of Harbin Medical University, Harbin, P.R. China; 2 Department of Ophthalmology, No.2 Hospital of Xiamen, Fujian, P.R. China; University of Colorado Denver School of Medicine, UNITED STATES

## Abstract

Posterior capsule opacification (PCO) is the most common complication that causes visual decrease after extracapsular cataract surgery. The primary cause of PCO formation is the proliferation of the residual lens epithelial cells (LECs). The mammalian target of rapamycin (mTOR) plays an important role in the growth and migration of LECs. In the current study, we used small interfering RNA (siRNA) to specifically attenuate mTOR in human lens epithelial B3 cells (HLE B3). We aimed to examine the effect of mTOR-siRNA on the proliferation, migration and epithelial-to-mesenchymal transition (EMT) of HLE B3 cells and explore the underlying mechanisms. The mTOR-siRNA was transfected into HLE B3 cells using lipofectamine 2000. The mRNA and protein levels of mTOR were examined to confirm the efficiency of mTOR-siRNA. The levels of mRNA and protein as well as the activity of mTOR down-stream effectors p70 ribosomal protein S6 kinase (p70S6K) and protein kinase B (PKB, AKT) were examined using real-time PCR or Western blot, respectively. The cell proliferation was determined using cell counting kit (CCK) 8 and cell growth curve assay. The cell migration was examined using Transwell system and Scratch assay. MTOR-siRNA effectively eliminated mTOR mRNA and protein. The proliferation and migration were significantly suppressed by mTOR-siRNA transfection. mTOR-siRNA reduced the mRNA of p70S6K and AKT in a time-dependent manner. Furthermore, the phosphorylation of p70S6K and AKT was decreased by mTOR-siRNA. MTOR-siRNA also eliminated the formation of mTORC1 and mTORC2 protein complex and blocked the transforming growth factor (TGF)-β-induced EMT. Our results suggested that mTOR-siRNA could effectively inhibit the proliferation, migration and EMT of HLE B3 cells through the inhibition of p70S6K and AKT. These results indicated that mTOR-siRNA might be an effective agent inhibiting HLE cells growth and EMT following cataract surgery and provide an alternative therapy for preventing PCO.

## Introduction

Posterior capsule opacification (PCO), also known as after-cataract, is the most common complication and the primary reason for decreased visual acuity after extracapsular cataract surgery [1]. The primary cause of PCO formation is the proliferation of the residual lens epithelial cells (LECs). The leftover LECs started to proliferate within only a few hours after the cataract surgery and then migrated across the posterior capsule. The LECs underwent lens fiber regeneration and epithelial- mesenchymal transition (EMT) [2]. Therefore, a large number of studies have been taken to explore an efficient way to inhibit the proliferation, migration and EMT of LECs in order to prevent the formation of PCO.

Several lines of evidence indicated that the phosphatidylinositol 3-kinase (PI3K)/the mammalian target of rapamycin (mTOR) signalling pathway may be involved in the LECs proliferation and migration. MTOR, also known as FRAP (FKBP12-rapamcyin-associated protein), RAFT1 (rapamycin and FKBP12 target), RAPT1 (rapamycin target 1), or SEP (sirolimus effector protein), is a highly conserved serine/threonine kinase in the mammalian cells. MTOR plays a crucial role in cell-cycle progression, protein synthesis, angiogenesis, and apoptosis [3,4]. Intracellular mTOR forms two distinct protein complexes (mTORC): mTORC1 and mTORC2 [5]. Although both mTORC1 and mTORC2 are able to modulate proliferation and migration, they exert their functions via distinct signalling pathways. MTORC1 activates ribosomal S6 kinases (S6K1 and S6K2) and eukaryotic initiation factor 4E (eIF4E) to regulate cell-cycle progression and protein synthesis [6,7], whereas mTORC2 phosphorylates protein kinase B (PKB, AKT) at serine 473 to modulate cell differentiation, proliferation, invasion, and glucose metabolism [8–10].

Our group recently showed that rapamycin, an mTOR inhibitor, inhibited the proliferation of LECs [11]. Accumulating evidence indicates that mTOR signalling is also involved in EMT of human lens epithelial (HLE) cells [12,13]. Transforming growth factor-β (TGF-β)-induced EMT in HLE cells requires the activation of mTORC2 pathways [13]. Given that rapamycin does not target mTORC2 signalling pathway but mTORC1, we considered reducing the mTOR levels using small interfering RNA (siRNA). We predicted that attenuating the mTOR would effectively reduce the formation of mTORC1 and mTORC2 and thus improve the efficiency of preventing the proliferation, migration and EMT of LECs.

The aim of this study was to evaluate the potency of siRNA to transiently inhibit mTOR expression in HLE B3 cells and to examine its effects on cell proliferation, migration and EMT. We also aimed to examine whether mTOR-siRNA inhibits mTORC1 and mTORC2 signalling pathways.

## Materials and Methods

### Cell Culture

HLE B3 cells were purchased from the American Type Culture Collection (ATCC, Manassas, VA, USA), grown in Dulbecco’s modified Eagle’s medium (DMEM, Hyclone, Beijing, China) supplemented with 15% premium fetal bovine serum (FBS) (Biological Industries, Israel), 50 U/ml of penicillin and 50 μg/ml streptomycin (Hyclone, Beijing, China). Cells were maintained at 37°C in a humidified 5% CO_2_ atmosphere.

### SiRNA Transfection

MTOR-siRNA and non-silencing siRNA were purchased from Santa Cruz (Santa Cruz Biotechnology, USA). Transient transfection of siRNA was performed using lipofectamine transfection reagent 2000 (Invitrogen, Carlsbad, CA), according to the manufacturer’s protocol. Control cells were mock transfected with lipofectamine 2000 reagent only. HLE B3 cells (1,200,000 cells per well) were seeded in a 6-well plate and cultured for 16 hours until cells reached 60%-70% confluence. The complete culture medium was replaced with serum-free and antibiotic-free medium at 1 hour before transfection. The cells were incubated with transfection mixtures containing 30 nM of mTOR-siRNA or non-silencing RNA for 5 hours, and then the medium was replaced with full culture medium.

### Cell Count Kit (CCK) 8 Proliferation Assay

To determine the proliferative ability of cells, HLE B3 cells (10,000 cell/well) were cultured in 96-well flat bottom plates in a triplicate pattern. The cells were firstly transfected with mTOR-siRNA or non-silencing siRNA. After 24, 48 and 72 hours of transfection, 10 μl CCK8 (Beyotime, Nanjing, China) solution was added into each well which contained 100 μl culture media and incubated for 2 hours at 37°C. Finally, the optical density value of each well was measured by absorbance at 490 nm in a microplate reader (Eppendorf, Hamburg, Germany).

### Cell Growth Curve Assay

To determine the proliferation rate, 8×10^4^ cells were plated in a 12-well plate (triplicates for each time point) and cultured for 24 hours until cells reached about 70% confluence. Cells were then transfected with mTOR-siRNA or non-silencing siRNA. After 24, 48 and 72 hours of transfection, cells were trypsinized and counted four times with a hemocytometer (QiuJing Inc., Shanghai, China). HLE B3 cells were also treated with Rapamycin (80 nmol/L as final concentration, Sigma Chemical Co, St. Louis Mo) for 24, 48 and 72 hours and the cell number was counted.

### Cell Migration Assay and Scratch Assay

Cell motility was determined *in vitro* firstly using a Transwell chamber (BD Biosciences, New Jersey, USA). Twenty-four hours after being transfected with mTOR-siRNA or non-silencing RNA, cells were trypsinized and placed into the upper wells of the Boyden chamber (100,000 cells per well) in 100 μl DMEM with 20% FBS. In the lower chamber, 600 μl DMEM containing 10% FBS was added. Cells in the Boyden chamber were incubated for 48 hours at 37°C in a 5% CO_2_ incubator. After non-migrated cells were scraped off, the membrane was fixed with methanol, stained with Hemacolor stain set (EM Industries, Inc, Gibbstown, NJ). Relative migration was based on the average number of cells on the underside of the membrane in four random images generated at 4 × magnification under microscope. Cell migration was analyzed using the Gel-Pro analyzer program (Media Cybernetics, Silver Spring, MD).

For the Scratch assay, 24 hours after mTOR-siRNA transfection, cells were trypsinized and seeded into 6 well dishes in standard growth medium. Twenty-four hours later, a scratch was made with a 200μl pipette tip and TGF-β2 (2 μg/ml as final concentration, BioVision, USA) was added into culture medium. Cell migration induced by TGF-β was examined at 48 hours. Cell images of 0 time point and 48 hours after scratch were taken with a digital camera connected to an inverted Olympus microscope (20 × magnification). The same visual field was marked and used at both time points. The gap area was measured using Image-Pro Plus software. Gap closure (%) = [Gap area (T48-T0)/Gap area T0] × 100% (T0 is the time that the scratch was made and T48 is the time of analysis).

### Quantitative Real-Time PCR

Total RNA was extracted with TRIzol (Life Technologies, Grand Island, NY, USA). First-strand complementary cDNA was generated using RevertAid First Strand cDNA Synthesis kit (TaKaRa, Dalian, China) according to manufacture’s protocol. 1μg RNA was used for cDNA synthesis. For PCR, 10 μl 2 × qPCR Mix, 0.5 μl each primer (10 μM), 2 μl cDNA and 7 μl double distilled H_2_O were added to a 0.2 ml PCR tube. Amplification conditions were as follows: 95°C for 10 minutes, 45 cycles of 95°C for 5 seconds and 60°C for 40 seconds. Rea-time PCR was performed using ABI 7500 Thermal cycler (Applied Biosystems, USA). The levels of genes of interest were standardized to the level of Actin. The real-time PCR primers are listed in Table 1.

**Table 1:** Primer sequences employed for real-time PCR.

Primer Name	Forward Sequence	Reverse Sequence
Actin	CTGAAGTACCCCATCGAGCAC	ATAGCACAGCCTGGATAGCAAC
mTOR	AAAACCTCAGCATCCAGAGATACGC	CATCAGAGTCAAGTGGTCATAGTCCG
p70S6K	AGTAAAGCATCCCTTCATCGTGG	TGATGTAAATGCCCCAAAGCC
AKT	ACGGGCACATTAAGATCACAGA	GCTTCTCATGGTCCTGGTTGTAG

### Western Blot

HLE B3 cells were transfected with mTOR-siRNA or non-silencing siRNA. After 72 hours, HLE B3 cells were lysed in radio immunoprecipitation assay (RIPA) lysis buffer (10 mM Tris-Cl, pH 8.0, 1 mM EDTA, 0.5 mM EGTA, 1% Triton-100, 0.1% sodium deoxycholate, 0.1% SDS and 140 mM NaCl) containing freshly prepared complete protease inhibitor cocktail and phosphatase inhibitor cocktail (Roche, Beijing, China). Equal amounts of total protein were loaded into each lane of a 12% SDS-PAGE gel and then electrotransferred to a nitrocellulose membrane (Invitrogen). The membrane was blocked with 5% skim milk in PBST (0.1% Tween 20) for 1 hour. The membranes were incubated with rabbit anti-human phosphorylated p70S6K (1:1000; CST, Danvers, MA, USA) or rabbit anti-phosphorylated AKT (CST) in 5% skim milk overnight, respectively. The membranes were incubated with a horseradish peroxidase (HRP) conjugated goat anti-rabbit antibody (1:1000; Santa Cruz Biotechnology, Santa Cruz, CA) for 60 min at room temperature. Protein expression was assessed by enhanced chemiluminescence and exposure to chemiluminescent film (Thermo Fisher Scientific, Massachusetts, USA). The membrane was then washed and incubated with mouse anti-human p70S6K or glyceraldehyde-3-Phosphate Dehydrogenase (GAPDH) (1:1000; KangChen Bio-tech, Shanghai, China). GAPDH was used as a loading control. Antibodies used were against Rictor (Abcam, Cambridge, England), Raptor (Abcam), mTOR (Cell Signaling Technology, Shanghai, China).

### Co-Immunoprecipitation (IP)

HLE B3 cells were transfected with mTOR-siRNA and cultured for 72 hours. Cells were lysed in RIPA buffer on ice for 5 minutes. The samples were sonicated on ice three times for 10 seconds each and then centrifuged at 14,000 × g for 10 minutes at 4°C. The supernatant was then incubated with primary antibody against mTOR with rocking overnight at 4°C. Twenty μl of Protein A agarose beads slurry (Thermo Fisher Scientific) were added into each sample and incubated for 3 hours at 4°C. The beads were then centrifuged for 30 seconds and washed with RIPA buffer for three times. The beads were resuspended in 2 × SDS sample buffer and the supernatant was subjected to Western blot.

### Statistical Analysis

Experiments were carried out in triplicate and repeated twice. Values were expressed as mean ± SEM. The data were analyzed by one-way ANOVA with Tukey’s post hoc test. Values *p* < 0.05 were considered statistically significant.

## Results

### MTOR-siRNA Attenuated mTOR Expression

**Fig 1:**
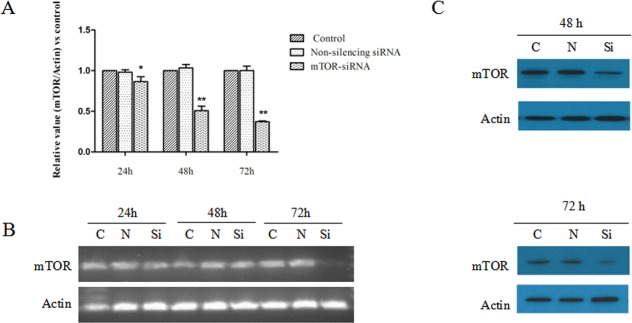
The effect of mTOR-siRNA on the levels of mTOR mRNA and protein. HLE B3 cells were treated with mTOR-siRNA, non-silencing siRNA or control (transfection reagent only), and harvested after 24 h, 48 h and 72 h of transfection. (**A**) MTOR mRNA was quantified by real-time PCR. The values of mTOR were normalized to Actin and then normalized to control relative value. (**B**) Representative agarose gel images of the real-time PCR products. (**C**) MTOR protein levels were examined by Western blot. (Data = Mean ± SEM, *p<0.05, **p<0.01, compared with the control groups).

The efficiency of mTOR-siRNA was examined by real-time PCR and Western blot. We demonstrated a decrease in the total mTOR mRNA in HLE B3 cells treated with mTOR-siRNAs relative to controls at 24, 48 and 72 hours after transfection, respectively (14.6%, 49.3% and 62.8% decrease, respectively) (**Fig 1A and 1B**). The results suggested that the expression of mTOR mRNA was effectively eliminated by mTOR-siRNA in a time-dependent manner. The mTOR protein was also dramatically reduced by mTOR-siRNA at 48 hours and 72 hours after transfection (**Fig 1C**).

### MTOR-siRNA Decreased Cell Proliferation

**Fig 2:**
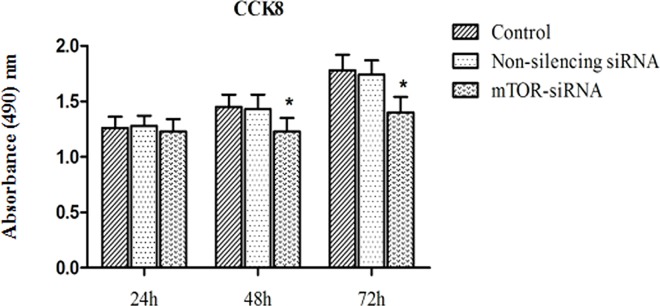
The effect of mTOR-siRNA on HLE B3 proliferation. CCK8 assay was used to determine HLE B3 proliferation. MTOR-siRNA significantly reduced cell proliferation at 48 hours of transfection and this effect was enhanced at 72 hours of transfection. (Data = Mean ± SEM, *p<0.05, compared with the control groups).

CCK8 assay was used to examine the effect of mTOR-siRNA on the cell proliferation. MTOR-siRNA significantly inhibited the proliferation of HLE B3 cells by 15.2% and 21.3%, after 48 hours and 72 hours of transfection, respectively (**Fig 2**).

**Fig 3:**
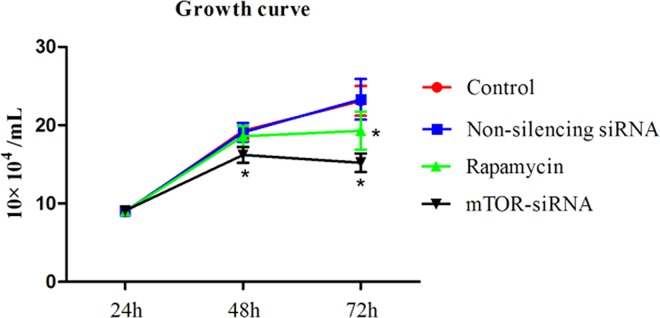
The HLE B3 cells growth curve in the presence of mTOR-siRNA and rapamycin. mTOR-siRNA inhibited cell growth at 48 hours of transfection and this effect was dramatically enlarged at 72 hours of transfection. Rapamycin significantly reduced cell growth at 72 hours of transfection. (Data = Mean ± SEM, *p < 0.05).

The cell proliferation rate was examined using a cell growth curve. We observed that the cell number drastically increased when cells were treated with mock reagent and non-silencing siRNA. Non-silencing siRNA did not alter the growth rate, compared with the control. However, the cell number was significantly reduced by mTOR-siRNA treatment. Compared to the control, 16.1% and 34.2% decrease were observed in mTOR-siRNA treated cells at 48 hours and 72 hours post-transfection (16.1% and 34.2% decrease, respectively) (**Fig 3**). Rapamycin, an inhibitor of mTOR (mTORC1 pathway) trimmed off 20.2% cell growth at 72 hours after transfection.

### MTOR-siRNA Reduced Cell Mobility

**Fig 4:**
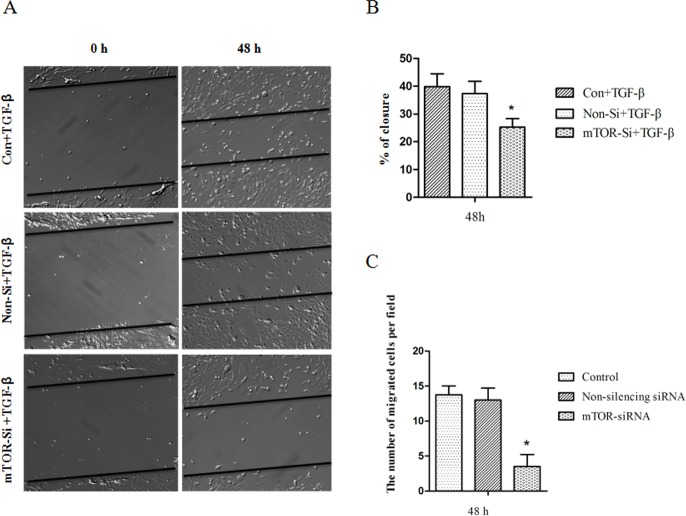
The inhibition of cell migration by mTOR-siRNA. (**A**) Representative images were taken from Scratch assay. (**B**) TGF-β-induced cell migration was blocked by mTOR-siRNA. The Gap closure was reduced by mTOR-siRNA. (**C**) Cell migration was assessed using the Byoden chamber in the absence of TGF-β. The mTOR-siRNA transfected HLE B3 cells were seeded into the Boyden chamber and incubated for 48 hours. The cell migration was significantly reduced by mTOR-siRNA at 48 hours. (Data = Mean ± SEM, *p < 0.05).

The migration of HLE B3 cells was firstly examined using a classical Scratch assay. A scratch was made at 24 hours of siRNA transfection and TGF-β was applied to induce migration. After 48 hours, cell mobility significantly reduced in mTOR-siRNA treated cells compared with control groups (**Fig 4A** and 4**B**). We also tested the number of migrated cells using Boyden chamber in the absence of TGF-β. Twenty-four hours after mTOR-siRNA transfection, HLE B3 cells were seeded into the chamber and the migrated cells were counted at 48 hours of migration. mTOR-siRNA significantly decreased the number of cells migrating into lower chamber (74.5% decrease) (**Fig 4C**).

### The Influence of mTOR-siRNA on the Levels of p70S6K

**Fig 5:**
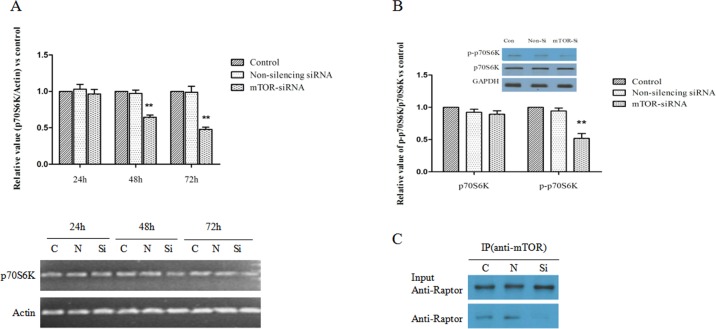
MTOR-siRNA reduced the formation of Raptor/mTOR complex and inhibited the phosphorylation of p70S6K. (**A**) mTOR-siRNA significantly down-regulated p70S6K mRNA expression at 48 hours of transfection, and this effect was enlarged at 72 hours of transfection. The values of p70S6K mRNA were normalized to Actin mRNA and then normalized to control relative value. (**B**) The phosphorylation of p70S6K was reduced by mTOR-siRNA after 72 hours of transfection. (**C**) The interaction of Raptor and mTOR were assessed using co-immunoprecipitation assay. In the anti-mTOR antibody precipitates, the levels of Raptor were drastically reduced by mTOR-siRNA. (Data = Mean ± SEM, **p<0.01, compared with the non-silencing siRNA).

As mTOR-siRNA dramatically reduced the mTOR in HLE B3 cells, we then evaluated its effects on mTORC1 and mTORC2 pathways. We firstly examined p70S6K, a downstream effector of mTOR in mTORC1 pathway. Real-time PCR results showed that mTOR-siRNA significantly reduced the mRNA levels of p70S6K. MTOR-siRNA transfection led to 3.5%, 35.4% and 51.6% reduction of p70S6K mRNA at 24, 48 and 72 hours of transfection, respectively (**Fig 5A**). Surprisingly, we did not observe a down-regulation of p70S6K protein after mTOR-siRNA treatment. However, the phosphorylation of p70S6K was reduced by 48.1% at 72 hours after mTOR-siRNA transfection in comparison with control group (**Fig 5B**). Total level of Raptor, a key protein in mTORC1 complex did not alter, but the interaction of Raptor and mTOR were dramatically decreased by mTOR-siRNA treatment (**Fig 5C**).

### The Influence of mTOR-siRNA on the Levels of AKT Expression and mTORC2 Complex Formation

**Fig 6:**
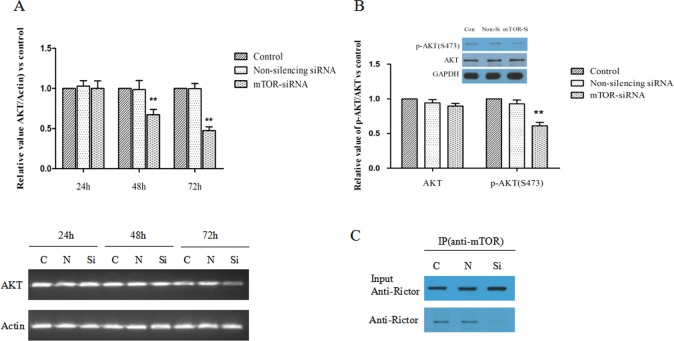
MTOR-siRNA eliminated the protein interaction of Rictor and mTOR protein and inhibited the phosphorylation of AKT. (**A**) HLE B3 cells were treated with mTOR-siRNA, non-silencing siRNA or control and harvested after 24, 48 and 72 hours of transfection. The levels of mRNA were determined by quantitative real-time PCR. The values of AKT mRNA were normalized to Actin mRNA and then normalized to the control value. (**B**) The phosphorylation of AKT was reduced by mTOR-siRNA after 72 hours of transfection. (**C**) The interaction of Rictor and mTOR proteins was assessed by co-immunoprecipitation using an anti-mTOR antibody. The precipitates were examined by western blot with anti-Rictor antibody. In the mTOR antibody precipitates, Rictor was undetectable in the samples treated with mTOR-siRNA. (Data = Mean ± SEM, **p<0.01, compared with the non-silencing siRNA).

We then examined AKT, a downstream effector of mTORC2 pathways. Results showed that AKT mRNA expression was significantly down-regulated at 48 and 72 hours after mTOR-siRNA transfection (32.8% and 52.7% decrease, respectively) (**Fig 6A**). The protein level of AKT was not altered by mTOR-siRNA at 72 hours after transfection. However, we observed a significant reduction of the phosphorylated AKT (39.0% decrease) in mTOR-siRNA transfected cells (**Fig 6B**). The total Rictor, a key protein in mTORC2 complex did not show difference in mTOR-siRNA treated cells. Co-IP analysis showed that Rictor was almost undetectable in mTOR antibody precipitates in the samples treated with mTOR-siRNA, indicating that the interaction of Rictor and mTOR proteins was reduced (**Fig 6C**). These results suggested that mTOR-siRNA might inhibit both mTORC1 and mTORC2 pathway by decreasing the activity of p70S6K and AKT.

### The Effect of mTOR-siRNA on EMT Induced by TGF-β

**Fig 7:**
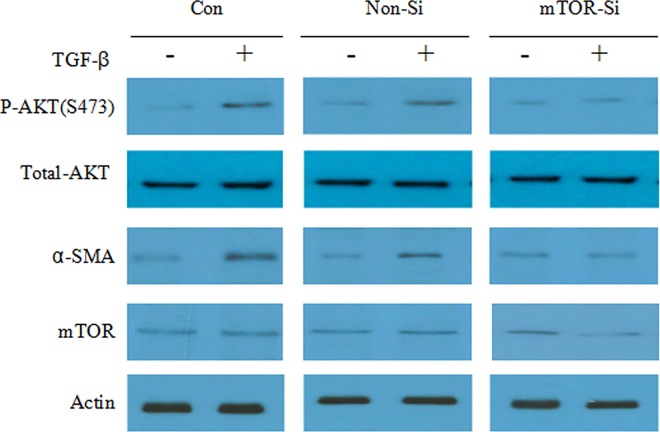
MTOR-siRNA blocked EMT induced by TGF-β. HLE B3 cells were transfected with mTOR-siRNA and 24 hours later cells were treated with TGF-β for 48 hours. Cells were then lysed and subjected to Western blot.

EMT is one of the key steps in PCO formation, we therefore examined whether mTOR-siRNA could inhibit the EMT. We used TGF-β to induce EMT of HLE B3 cells and examined the level of α-smooth muscle actin (α-SMA), which is expressed once HLE trans-differentiated into myofibrolast-like cells. HLE B3 cells transfected with mTOR-siRNA were treated with TGF-β 24 hours post-transfection for 48 hours. TGF-β promoted the phosphorylation of AKT, indicating the involvement of mTORC2 signaling. TGF-β also increased the level of α-SMA in comparison with control treatment. However, this effect was eliminated by mTOR-siRNA treatment (**Fig 7**).

## Discussion

The main findings in this study are: 1) mTOR-siRNA effectively reduced mTOR levels in HLE B3 cells; 2) Attenuating mTOR dramatically inhibited HLE B3 proliferation; 3) The activities of p70S6K and AKT, downstream effectors of mTORC1 and mTORC2, were reduced by mTOR interference; 4) TGF-β-induced EMT was blocked by mTOR inhibition.

PCO is the most common postoperative complication of cataract surgery, which causes visual loss. Residual LECs at the equator and under the anterior lens capsule can proliferate after surgery and result in the formation of PCO [14]. Our previous study reported that rapamycin reduced the proliferation of rabbit LECs by inhibiting mTORC1 pathway [11]. In the current study, we showed that mTOR-siRNA inhibited both mTORC1 and mTORC2 pathways and dramatically reduced the proliferation, migration and EMT of HLE B3 cells.

Several clinical trials of mTOR targeted-therapeutic are currently underway in cancer clinics. *In vitro* studies also found that mTOR-siRNA suppressed the growth of esophageal squamous cell carcinoma (ESCC), human colon cancer cells and non-small cell lung cancer cells [15–18]. Our current study showed that mTOR-siRNA targeted at both mTORC1 and mTORC2 signaling pathways. MTORC1 is formed by regulatory associated protein of mTOR (Raptor), mTOR associated protein LST8 homolog (mLST8, also known as GbL) and DEP domain containing mTOR-interacting protein (Deptor). Rapamycin inhibits mTORC1 formation and reduces the phosphorylation of p70S6K and eukaryotic initiation factor 4E (eIF4E) binding protein 1 (4E-BP1) [19]. Both p70S6K and eIF4E are crucial in cell growth. Downregulation of p70S6K or eIF4E led to a reduction in translation of a subset of genes that are required for protein synthesis and cell growth [20–23]. Our results firstly showed that p70S6K mRNA was reduced by mTOR-siRNA. Besides, we showed that the phosphorylation of p70S6K was reduced by mTOR-siRNA. These results are consistent with the study of Hou et al that mTOR-siRNA not only regulated the phosphorylation of p70S6K but also modulated its mRNA expression [15]. In addition, we observed that mTOR-siRNA reduced the interaction between mTOR and Raptor. These results suggested that mTOR-siRNA regulates mTORC1 pathway.

MTORC2 included Raptor-independent companion of mTOR (Rictor), Sin1, GbL and Deptor. We also showed that mTOR-siRNA reduced AKT expression and AKT phosphorylation. Furthermore, we found that mTOR-siRNA reduced the interaction between mTOR and Rictor. AKT is the central molecule in the PI3K/AKT/mTOR pathway, activating and modulating numerous downstream targets. AKT can stimulate protein synthesis and cell growth by activating mTOR through inhibition of the Tuberous sclerosis complex (TSC1/2) and modulating cell proliferation by inactivating cell cycle inhibitors [24–28]. It has been described that mTORC2 participates in cell survival and proliferation in part through controlling AKT activity by phosphorylation of AKT at serine-473 [29]. Taken together, our results strongly suggested that mTOR-siRNA is able to inhibit both mTORC1 and mTORC2 pathway and therefore leads to profound inhibition of cell proliferation and migration. In addition, we found that mTOR-siRNA also blocked the TGF-β-induced AKT phosphorylation and α-SMA expression, suggesting that mTOR-siRNA potentially inhibits EMT.

RNAi is a new tool for gene silencing, using antisense nucleic acids and gene targeting [30,31]. RNAi was originally recognized as an evolutionary conserved defence mechanism in higher eukaryotic cells, and this system can easily and effectively inhibit the expression of one specific gene [32]. We attenuated mTOR levels using RNAi technology, which lead to the inhibition of mTOR/p70S6K/AKT pathways. Given that mTOR-siRNA induced a profound decrease in LECs proliferation, migration and EMT, we could potentially find a more optimal method avoiding the PCO formation.

To the best of our knowledge, this is the first report that mTOR-siRNA transfection can effectively repress LECs proliferation, migration and EMT via inhibiting both mTORC1 and mTORC2 pathways. Further studies are warranted to examine the effect of mTOR-siRNA on the formation of PCO after cataract surgery *in vivo*. Nevertheless, based on our results, we presume that mTOR-siRNA might be an effective agent affecting the behavior of HLE cells following cataract surgery. Further work is required before this approach can be used for the treatment of human disease.
